# Activation of the SIRT1/p66shc antiapoptosis pathway via carnosic acid-induced inhibition of miR-34a protects rats against nonalcoholic fatty liver disease

**DOI:** 10.1038/cddis.2015.196

**Published:** 2015-07-23

**Authors:** W Shan, L Gao, W Zeng, Y Hu, G Wang, M Li, J Zhou, X Ma, X Tian, J Yao

**Affiliations:** 1 Department of Pharmacology, Dalian Medical University, Dalian, China; 2 Department of General Surgery, Second Affiliated Hospital, Dalian Medical University, Dalian, China

## Abstract

Recent studies have demonstrated that miR-34a expression is significantly upregulated and associated with apoptosis in nonalcoholic fatty liver disease (NAFLD). Carnosic acid (CA) is a novel antioxidant and a potential inhibitor of apoptosis in organ injury, including liver injury. This study aimed to investigate the signaling mechanisms underlying miR-34a expression and the antiapoptotic effect of CA in NAFLD. CA treatment significantly reduced the high-fat diet (HFD)-induced elevations in aminotransferase activity as well as in serum triglyceride (TG), total cholesterol (TC), low-density lipoprotein cholesterol (LDL-C) and malondialdehyde (MDA) levels but increased serum high-density lipoprotein cholesterol (HDL-C) and hepatic superoxide dismutase (SOD) levels. Moreover, CA treatment ameliorated the increase in cleaved caspase-3 caused by HFD exposure and completely reversed the HFD-induced decreases in manganese superoxide dismutase (MnSOD) and B-cell lymphoma-extra large expression. CA also counteracted the HFD- or palmitic acid (PA)-induced increases in caspase-3 and caspase-9 activity. Mechanistically, CA reversed the HFD- or PA-induced upregulation of miR-34a, which is the best-characterized regulator of SIRT1. Importantly, the decrease in miR-34a expression was closely associated with the activation of the SIRT1/p66shc pathway, which attenuates hepatocyte apoptosis in liver ischemia/reperfusion injury. A dual luciferase assay in L02 cells validated the modulation of SIRT1 by CA, which occurs at least partly via miR-34a. In addition, miR-34a overexpression was significantly counteracted by CA, which prevented the miR-34a-dependent repression of the SIRT1/p66shc pathway and apoptosis. Collectively, our results support a link between liver cell apoptosis and the miR-34a/SIRT1/p66shc pathway, which can be modulated by CA in NAFLD.

Nonalcoholic fatty liver disease (NAFLD) is the hepatic manifestation of metabolic syndrome, and it is currently a significant heath concern worldwide.^1^ The disease spectrum of NAFLD commonly encompasses mere steatosis, nonalcoholic steatohepatitis (NASH), fibrosis and cirrhosis.^2^ At present, the pathological mechanisms of NAFLD development are primarily ascribed to lipid metabolism disorders, oxidative stress and hepatocyte apoptosis, as interpreted by the ‘double-hit' hypothesis.^3^ Several studies have confirmed that hepatocyte apoptosis is a pivotal event in several types of liver injury, including NAFLD.^4, 5^ Therefore, a thorough understanding of the mechanisms that regulate apoptosis may be clinically relevant for preventing and treating NAFLD.^6^

In recent years, increasing attention has been directed at natural herbs for the treatment of metabolic diseases.^7^ *Rosmarinus officinalis L* (Lamiaceae) is a herbal plant that is extensively used by the food industry for its beneficial health properties.^8^ Carnosic acid (CA), which is one of the major phenolic compounds extracted from the leaf of this plant, exhibits various pharmacological properties, including anti-steatosis, antioxidant and antitumor activity.^9, 10, 11^ CA induces apoptosis in cancer cells by affecting the expression of genes that regulate apoptosis.^12, 13^ In addition to inducing apoptosis in most types of cancer cells, CA has been suggested to exert its protective effect against organ injury by inhibiting apoptosis. For example, CA decreased isoproterenol-induced myocardial lipid peroxidation and cardiomyocyte apoptosis.^14^ Furthermore, CA attenuated 6-hydroxydopamine-induced apoptosis in SH-SY5Y cells, and CA is a potential candidate for neuroprotection in Parkinson's disease.^15^ Owing to its antioxidant and antiapoptotic properties in renal cells, CA has a protective effect on cisplatin-induced experimental nephrotoxicity.^16^ Our laboratory reported that CA inhibited hepatic apoptosis induced by liver ischemia/reperfusion injury.^17^ However, the molecular mechanisms by which CA regulates apoptosis in NAFLD remain unknown.

MicroRNAs (miRNAs) are highly conserved, small, noncoding RNAs that regulate gene expression by binding to complementary sites on target transcripts and are important modulators of pathophysiology processes.^18^ miR-34a, a prime putative player that induces senescence, cell cycle arrest and apoptosis, has recently garnered attention because of its significance in metabolic diseases.^19, 20^ Several miRNAs have been identified as molecular targets of phenols underlying their biological effects.^21, 22^ Accordingly, our preliminary data suggested that miR-34a expression may be decreased in response to CA in models of NAFLD. Thus, we investigated the underlying mechanism by which the CA-induced decrease in miR-34a expression protects against NAFLD.

NAFLD is characterized by increased levels of free fatty acid (FFA) and free cholesterol, which are the main inducers of the mitochondrial apoptosis pathway in NAFLD.^5, 23, 24^ p66shc, an isoform of the shcA adapter molecule, is a redox enzyme that has been implicated in promoting mitochondrial oxidative signalings into apoptosis.^25^ Interestingly, when NASH is exacerbated in humans, p66shc expression increases; p66shc was reported to be biologically active in the proapoptotic cascade triggered by p53 in an animal model of NASH.^26^ In addition, p66^−/−^ mice displayed increased resistance to oxidative stress-induced apoptosis after long-term ethanol exposure.^27^ Despite these observations that indicate a critical role for p66shc in the pathophysiology of metabolic diseases, there is limited information regarding the mechanisms that negatively regulate p66shc expression. Our laboratory has reported that CA exerts an antiapoptotic effect during liver ischemia/reperfusion injury by activating the SIRT1/p66shc pathway.^17^ SIRT1, an NAD-dependent deacetylase, is the best-characterized direct target of miR-34a.^28^ In addition, the apoptotic pathway that is regulated by the miR-34a/SIRT1 axis is involved in numerous diseases.^28, 29, 30^ Therefore, we investigated whether the protective effect of CA against NAFLD involves miR-34a regulation and promotion of the SIRT1/p66shc pathway.

The aims of this study were as follows: (1) to test whether the signaling mechanisms underlying the proapoptotic activity of miR-34a are associated with the SIRT1/p66shc pathway in NAFLD; (2) to investigate whether CA activates SIRT1/p66shc by inhibiting miR-34a; and (3) to elucidate the role of the antiapoptotic effect of CA in protecting against NAFLD.

## Results

### CA attenuates HFD-induced liver injury and lipid accumulation

**Figure 1:**
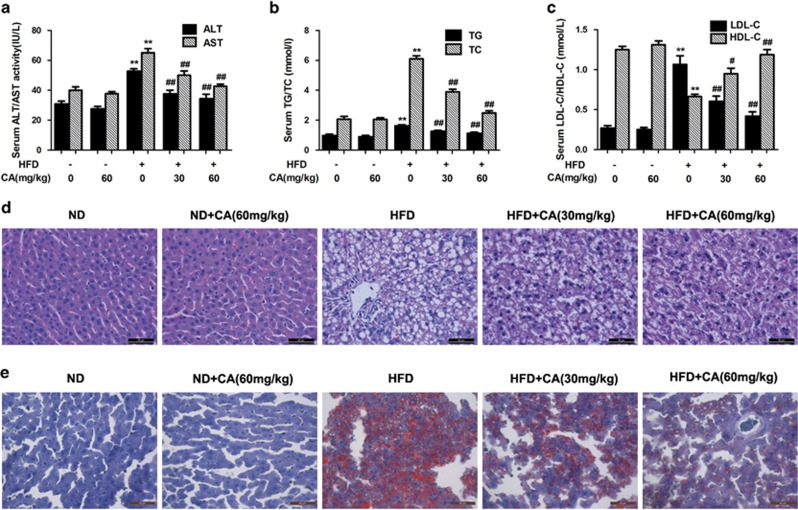
CA protects rats against HFD-induced liver injury and lipid accumulation. Rats were fed either a normal diet (ND) or a high-fat diet (HFD) alone or in combination with CA. (**a**) serum ALT and AST levels, (**b**) serum TG and TC levels, (**c**) serum HDL-C and LDL-C levels, (**d**) liver tissue sections were stained with H&E ( × 400), (**e**) liver tissue sections were stained with Sudan IV ( × 400). Scale bar, 50 *μ*m. The experimental groups subjected to H&E staining and Sudan IV staining were as follows: ND; ND+CA (60 mg/kg); HFD; HFD+CA (30 mg/kg); and HFD+CA (60 mg/kg). The data are presented as the mean±S.D. (*n*=10). **P<0.01 *versus* the ND group, ^*#*^*P*<0.05 *versus* the HFD group, ^*##*^*P*<0.01 *versus* the HFD group

The serum levels of ALT, AST, TG, TC, LDL-C and HDL-C were measured to ascertain the effect of CA on NAFLD.^31^ Serum ALT, AST, TG, TC and LDL-C levels were clearly increased and HDL-C levels were decreased in response to HFD compared with the control group (Figures 1a and c). However, CA treatment significantly abrogated the increases in ALT, AST, TG, TC and LDL-C levels and the decrease in HDL-C concentration in a dose-dependent manner (*P*<0.01), suggesting a protective effect of CA in HFD-fed rats. According to the H&E and Sudan IV staining, vehicle-treated rats exhibited no apparent abnormalities, but rats fed the HFD presented with nuclear pleomorphism and increased inflammatory infiltration, hepatocyte and hepatic cord degeneration, and lipid droplet accumulation. In contrast, CA treatment markedly alleviated the liver injury and attenuated the lipid accumulation caused by the HFD (Figures 1d and e). These results confirmed that CA protects rats against NAFLD.

### CA-mediated protection against NAFLD involves miR-34a downregulation and SIRT1/p66shc activation

**Figure 2:**
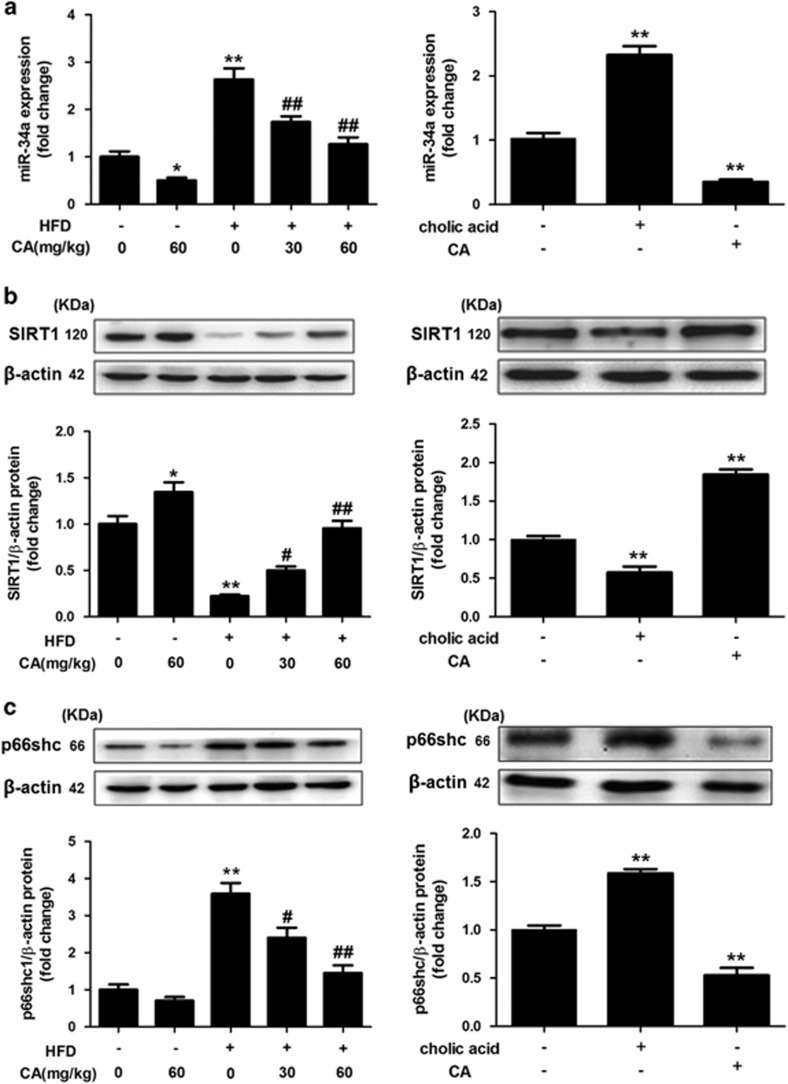
CA decreases miR-34a expression and activates the SIRT1/p66shc pathway in rat liver and L02 cells. (**a**) qRT-PCR analysis of miR-34a expression in the liver (left panel) and L02 cells (right panel). (**b**) SIRT1 protein expression in the liver (left panel) and L02 cells (right panel). (**c**) p66shc protein expression in the liver (left panel) and L02 cells (right panel). L02 cells were treated with CA, cholic acid or nothing. The data are presented as the mean±S.D. (*n*=3). *P<0.05 *versus* the ND group, **P<0.01 *versus* the ND group, ^*#*^*P*<0.05 *versus* the HFD group, ^*##*^*P*<0.01 *versus* the HFD group

To test the hypothesis that miR-34a downregulation and SIRT1/p66shc activation are associated with the CA-mediated attenuation of NAFLD, we measured the changes in the expression of miR-34a, SIRT1 and p66shc in response to CA *in vivo* and *in vitro*. As shown in the left panel of Figure 2a, miR-34a expression was increased in the HFD group compared with the ND group. Moreover, CA treatment significantly blunted the HFD-induced increase in miR-34a expression. As expected, SIRT1 expression was downregulated in the HFD group compared with the ND group, whereas p66shc protein expression was upregulated. However, CA abrogated the decrease in SIRT1 protein expression and the increase in p66shc expression in a dose-dependent manner (Figures 2b and c, left panel), which suggested SIRT1/p66shc pathway activation.

We next determined whether the *in vivo* results could be recapitulated in L02 cells. CA decreased miR-34a expression, but cholic acid was a strong inducer of miR-34a expression (Figure 2a, right panel).^32^ In agreement with the *in vivo* data, CA increased SIRT1 expression (Figure 2b, right panel) but significantly inhibited p66shc expression (Figures 2c, right panel). In contrast, cholic acid inhibited SIRT1 expression and increased p66shc expression (Figures 2b and c, right panel). Furthermore, CA had the same effect as antago-miR-34a (a miR-34a inhibitor), which blunted the overexpression of miR-34a in L02 cells exposed to PA (Figures 4a, c and d). Thus, these results suggested that miR-34a downregulation and SIRT1/p66shc activation are involved in the protective effect of CA against NAFLD.

### CA-mediated inhibition of miR-34a has an antiapoptotic effect in hepatocytes by targeting the SIRT1/p66shc pathway

**Figure 3:**
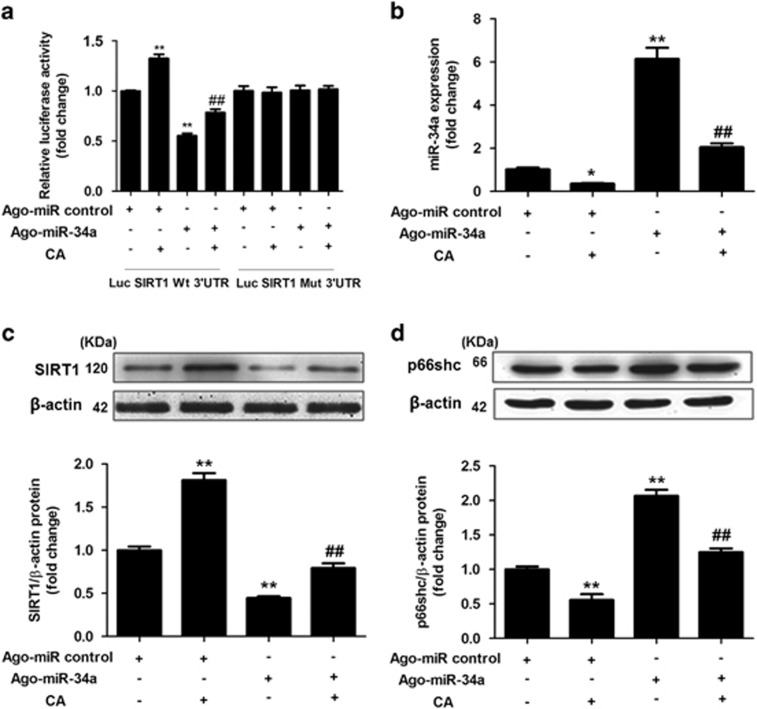
CA stimulates the SIRT1/p66shc antiapoptotic signaling pathway in a miR-34a-dependent manner. (**a**) CA modulates SIRT1 via miR-34a. L02 cells were transfected with wild-type or mutant SIRT1-3′-UTR luciferase constructs and with ago-miR-34a control or ago-miR-34a. After 24 h, the cells were exposed to 10 *μ*M CA for 12 h or were left untreated. (**b**–**d**) CA abrogates the loss of SIRT1 and the increase in p66shc following miR-34a overexpression. For functional analyses, L02 cells were transfected with the ago-miR-34a control or ago-miR-34a. After 24 h, the cells were exposed to 10 *μ*M CA for 12 h or were left untreated. (**b**) qRT-PCR analysis of miR-34a expression. (**c**) SIRT1 protein expression. (**d**) p66shc protein expression. The data are presented as the mean±S.D. (*n*=3). *P<0.05 *versus* the ago-miR control group, **P<0.01 *versus* the ago-miR control group, ^*##*^*P*<0.01 *versus* the ago-miR-34a group

To validate the modulation of SIRT1 by CA via miR-34a, we co-transfected ago-miR-34a and luciferase reporter plasmids containing the miR-34a-SIRT1 response element (wt-Luc-SIRT1) or a mutant miR-34a-SIRT1 response element (mut-Luc-SIRT1) in the presence or absence of CA. Although luciferase activity was significantly repressed by miR-34a overexpression, it increased in response to CA in both control and miR-34a-overexpressing cells (Figure 3a). However, these effects were not observed with the mutated SIRT1-3′-UTR (Figure 3a). These results revealed that CA increases SIRT1 expression at least partly in a miR-34a-dependent manner.

To further elucidate the cellular effects of CA on SIRT1, we transfected L02 cells with ago-miR-34a in the presence or absence of CA and determined the expression levels of miR-34a, SIRT1 and p66shc. Consistent with the luciferase assays, miR-34a expression was markedly increased after ago-miR-34a transfection compared with the control, whereas CA significantly diminished miR-34a expression (Figure 3b). In addition, miR-34a overexpression decreased SIRT1 expression by approximately twofold (*P*<0.01; Figure 3c) with a concomitant increase in p66shc expression (Figure 3d). Notably, CA reversed the loss of SIRT1 and the increase in p66shc induced by miR-34a overexpression (*P*<0.01). Thus, we concluded that CA may target the miR-34a/SIRT1/p66shc apoptotic signaling pathway in NAFLD.

**Figure 4:**
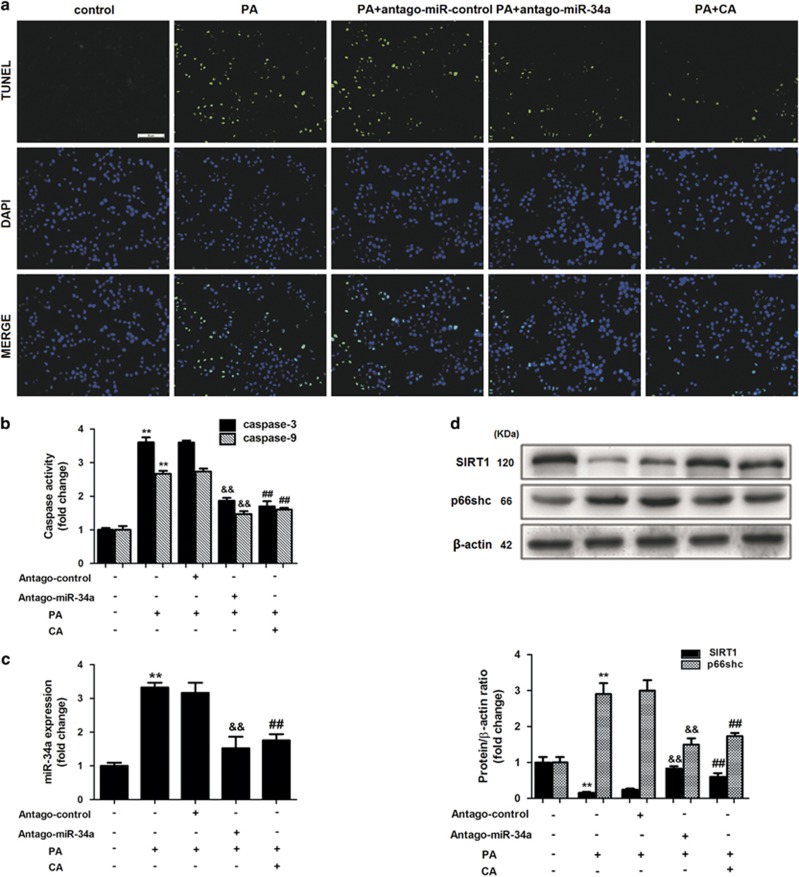
CA exerts an antiapoptotic effect through the miR-34a/SIRT1/p66shc pathway in L02 cells. L02 cells were treated with or without PA and then transfected with antago-miR-34a for 24 h or incubated with 10 *μ*M CA for 12 h. (**a**) TUNEL assay ( × 200), (**b**) Caspase-3 and caspase-9 activity, (**c**) miR-34a expression and (**d**) SIRT1 and p66shc protein expression. The data are presented as the mean±S.D. (*n*=3). *P<0.05 *versus* the ND group, **P<0.01 *versus* the control group, ^*##*^*P*<0.01 *versus* the PA group, ^*&&*^*P*<0.01 *versus* the PA+antago-miR control group

Finally, to evaluate whether the modulation of miR-34a/SIRT1/p66shc by CA had a significant impact on NAFLD, L02 cells were incubated with PA. Importantly, CA and antago-miR-34a proportionally inhibited FFA-induced apoptosis (Figures 4a and b). Consistent with the *in vivo* results, miR-34a was upregulated in PA-treated L02 cells, but miR-34a expression decreased after treatment with CA or antago-miR-34a (Figure 4c). Following PA-induced miR-34a overexpression, SIRT1 expression decreased, and p66shc expression increased. However, these effects were abrogated by CA (Figure 4d).

### Effects of CA on lipid peroxidation-induced apoptosis

**Figure 5:**
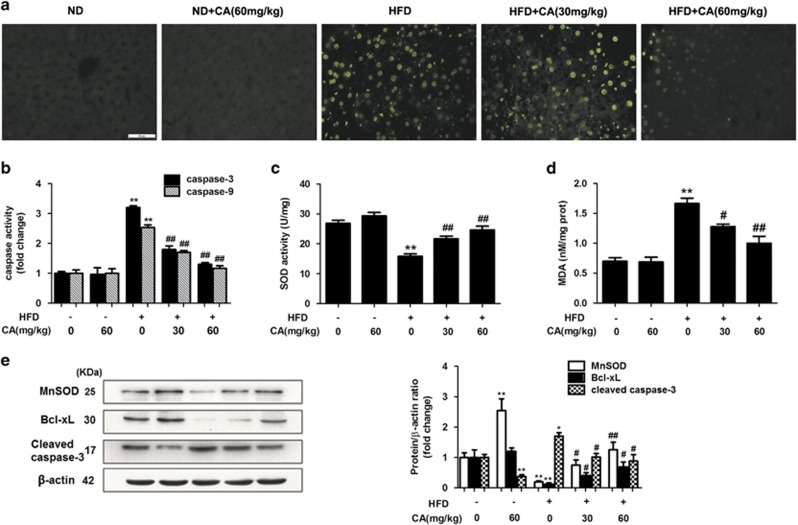
CA has an antiapoptotic effect in NAFLD. (**a**) Representative TUNEL staining ( × 400). Liver tissues were subjected to the TUNEL assay and imaged by fluorescent microscopy. The data are presented as the mean±S.D. of 10–12 frames/group from three or four animals/group. The groups were as follows: ND; ND+CA (60 mg/kg); HFD; HFD+CA (30 mg/kg); and HFD+CA (60 mg/kg). (**b**) Caspase-3 and caspase-9 activity in the liver. (**c**) Hepatic SOD activity. (**d**) Hepatic MDA levels. (**e**) The expression of MnSOD, Bcl-xL and cleaved caspase-3 was evaluated by western blotting (*n*=3). *P<0.05 *versus* the ND group, **P<0.01 *versus* the ND group, ^#^*P*<0.05 *versus* the HFD group, ^##^*P*<0.01 *versus* the HFD group

To evaluate the lipid peroxidation state of the liver of rats fed a HFD, we measured the levels of malondialdehyde (MDA), SOD and manganese superoxide dismutase (MnSOD) in the liver. HFD-fed rats displayed high levels of MDA and decreased levels of MnSOD and SOD compared with the ND group. In contrast, CA significantly reduced MDA levels and increased SOD levels and MnSOD expression in a dose-dependent manner (Figures 5c and e). Lipid peroxidation produces excess ROS, and p66shc amplifies ROS-induced apoptosis.^25^ Figures 5a and b illustrate the significant hepatocyte apoptosis in the HFD group; hepatocyte apoptosis was significantly improved in the CA-treated group. In addition, HFD increased cleaved caspase-3 levels but decreased B-cell lymphoma-extra large (Bcl-xL) expression compared with the control. Moreover, CA treatment resulted in increased Bcl-xL accumulation but decreased cleaved caspase-3 levels (Figure 5e). These results suggested that the effect of CA on NAFLD may be related to inhibiting hepatocyte apoptosis at least partly by modulating the miR-34a /SIRT1/p66shc pathway.

## Discussion

Apoptosis has been widely suggested to have a determinant role in the pathogenesis of NAFLD. Consequently, potential endogenous modulators of apoptosis may represent new tools for therapeutic intervention. CA, one of the major phenolic compounds extracted from *R. officinalis L* (Lamiaceae), is a well-established antioxidant and anti-adipogenic agent.^9, 10, 33^ Recently, CA was reported to regulate the expression of lipolysis-related genes, such as CPT1, to affect fatty-acid metabolism in C57BL/6J-ob/ob mice.^33^ In addition, CA has potential as an adjunct to cancer chemotherapy, primarily because of to its ability to induce apoptosis and inhibit the proliferation and migration of cancer cells.^34, 35^ Several studies have suggested that CA might be beneficial for treating certain diseases by inhibiting apoptosis. For example, CA protects SH-SY5Y cells, myocardial cells, renal cells and hepatocytes against injury by attenuating apoptosis.^14, 15, 16, 17^ However, the mechanisms by which CA regulates hepatocyte apoptosis in NAFLD remain unknown. In our study, we successfully generated a HFD-induced rat model of NAFLD, which exhibited liver injury and lipid accumulation (Figures 1 and 5c). HFD-fed rats and PA-treated hepatocytes displayed increased hepatocyte apoptosis. Moreover, CA alleviated liver injury, lipid accumulation and hepatic apoptosis. We further elucidated the molecular mechanisms involved in the protective effects of CA against NAFLD.

Recently, miRNAs have emerged as regulators of a wide range of biological process.^36^ Overexpression and silencing of miRNAs are common phenomena that participate in the pathogenesis of specific diseases, including metabolic diseases. Overexpression of miR-34a has been suggested to significantly increase lipid accumulation and FFA-induced apoptosis in cultured primary rat hepatocytes.^32^ In addition, miR-34a overexpression in lean mice resulted in obesity-related outcomes, and conversely, antagonism of miR-34a by LNA-modified ONs in diet-induced obese mice alleviated steatosis, inflammation and glucose intolerance.^37^ Interestingly, increased miR-34a levels have been reported in serum from NAFLD patients, and the expression of miR-34a in human liver significantly increases with NAFLD severity.^32, 38^ In this regard, HFD-fed rats exhibited increased miR-34a expression, lipid accumulation and apoptosis. However, CA treatment markedly alleviated the NAFLD induced by HFD in rats and diminished the increase in miR-34a expression, thereby indicating that CA may have the same effect as silencing miR-34a with LNA-modified ONs to protect rats against NAFLD. We investigated this possibility in the L02 cell line. As expected, CA diminished miR-34a expression and elicited the same effect as antago-miR-34a (miR-34a inhibitor) in the cell model. Thus, miR-34a represents a potential therapeutic target for CA in the treatment of NAFLD.

SIRT1, an NAD-dependent deacetylase, has a critical role in metabolic diseases, including NAFLD. Mice with a hepatocyte-specific deletion of SIRT1 display increased hepatic steatosis and inflammation.^39^ SIRT1 plasma levels were reported to exhibit an inverse correlation with liver steatosis in obese patients.^40^ Abundant data have implicated SIRT1 in the regulation of energy metabolism, inflammation, oxidative stress and apoptosis via targeting different genes in the liver.^39, 41^ SIRT1 can affect the promoter of its target gene, p66shc, through deacetylation of histone H3 lysine 9.^42^ Moreover, CA-mediated activation of the SIRT1/p66shc pathway has been suggested to protect rats against apoptosis in liver ischemia/reperfusion injury.^17^ Consistent with these observations, we found that CA treatment augmented SIRT1 expression and reversed the upregulation of p66shc caused by HFD or PA in a dose-dependent manner. These results suggested that SIRT1 suppresses p66shc expression during NAFLD and that activation of the SIRT1/p66shc pathway is involved in the hepatoprotective effect of CA in NAFLD.

Several phenols have been suggested to affect the expression of miRNAs and consequently regulate miRNA target genes.^21, 32^ Because SIRT1 is the best-characterized direct target of miR-34a, we investigated the relationship between the CA-induced downregulation of miR-34a and SIRT1/p66shc pathway activation. The dual luciferase assay revealed that miR-34a overexpression significantly decreased SIRT1 expression; no changes were observed in cells that were transfected with the mutant plasmid, suggesting that miR-34a directly binds to and downregulates SIRT1. Furthermore, CA reversed the loss of SIRT1 and the increase in p66shc levels induced by miR-34a overexpression. These results were not observed in cells transfected with the mutant plasmid. Together, these data suggested that SIRT1 is a target of miR-34a and that CA-mediated inhibition of miR-34a confers protection against NAFLD, which is related to the activation of the SIRT1/p66shc signaling pathway.

NAFLD presents as an imbalance in FFA metabolism and synthesis. ROS production and peroxidation resulting from FFA metabolism via oxidation trigger mitochondrial damage and apoptosis.^5, 23, 24^ p66shc has been suggested to mediate mitochondrial cell death pathways by increasing lipid peroxidation-induced apoptosis.^25^ Mice lacking p66shc were reported to exhibit increased resistance to ethanol-induced mitochondrial ROS generation and liver cell damage.^27^ Tomita K *et al.*^26^ demonstrated that p66shc was biologically active in the proapoptotic cascade triggered by p53 in an animal model of NASH. Our laboratory further revealed that p66shc is a target of SIRT1 in hepatocytes and that SIRT1-mediated inhibition of p66shc attenuates oxidative stress and apoptosis during liver injury in mice and rats.^17, 43^ Together with our data, these findings indicate that a miR-34a/SIRT1/p66shc-dependent mechanism targeted by CA is involved in regulating oxidative stress and apoptosis in NAFLD. Our data demonstrated that CA increases the HFD-induced downregulation of SOD and MnSOD, the primary superoxide scavenger, but decreases the levels of MDA, an indicator of lipid peroxidation (Figures 5d and e). Moreover, CA attenuated hepatocyte apoptosis and blunted the HFD-induced upregulation of cleaved caspase-3 and downregulation of Bcl-xl (Figures 5a, b and e). We and others have demonstrated that mechanisms that negatively regulate p66shc are beneficial for NAFLD. In addition to p53/p66shc signaling, we suggest that the miR-34a/SIRT1/p66shc-mediated proapoptotic pathway has a pivotal role in NAFLD. Our findings showed that CA exerts an antiapoptotic effect in NAFLD through the miR-34a/SIRT1/p66shc signaling axis.

In summary, the present study revealed for the first time that CA has a protective effect against hepatocyte apoptosis in NAFLD. The protective effects of CA are associated with the downregulation of miR-34a expression and are accompanied by SIRT1/p66shc signaling pathway activation, resulting in a significant reduction in cleaved caspase-3 levels and in the upregulation of Bcl-xL. These results indicate that CA confers protection against NAFLD, at least in part, by inhibiting the miR-34a/SIRT1/p66shc pathway. Therefore, the miR-34a/SIRT1/p66shc proapoptotic pathway may represent an attractive pharmacological target for the development of new drugs to impede the progression of NAFLD.

## Materials and methods

### Experimental animals and reagents

Male Sprague-Dawley rats ranging from 180 to 220 g were obtained from the Experimental Animal Center of Dalian Medical University (Dalian, China). CA (98% purity), which was extracted from *R. officinalis*, was purchased from Shanghai Winherb Medical Science Co., Ltd. (Shanghai, China) and dissolved in olive oil. The high-fat diet (HFD) contained several compounds that provide energy, as previously described.^44, 45^ Fifty experimental rats were randomly divided into five groups: (1) control; (2) control+CA (60 mg/kg/day); (3) HFD; (4) HFD+CA (30 mg/kg/day); and (5) HFD+CA (60 mg/kg/day). CA and olive oil were administered by gavage every day for 10 weeks. At the end of the experiment, blood and liver samples were harvested for analysis. All the procedures were performed in compliance with the Institute's guidelines and with the Guide for the Care and Use of Laboratory Animals. The study was approved by the institutional animal care committee of Dalian Medical University.

### Cell culture and treatment

The human L02^46^ hepatic cell line was obtained from China Cell Culture Center (Shanghai, China). The cells were grown in 1640 containing 10% (v/v) fetal bovine serum (FBS) in a humidified incubator with 5% CO2 at 37 °C. Both 1640 and FBS are Invitrogen products that were purchased from Life Biotechnologies (Carlsbad, CA, USA). Cholic acid (98% purity) and palmitic acid (PA; 98% purity) were purchased from Sigma-Aldrich (St. Louis, MO, USA). When indicated, cells were treated with 10 *μ*M CA, 10 *μ*M cholic acid, or neither (control) for 12 h before processing for total protein and RNA extraction.^32^ For the experiments, L02 cells were transfected with 10 nM ago-miR-34a (GenePharma, Shanghai, China) or ago-miR-negative control using Lipofectamine 2000 (Invitrogen). After 24 h, the cells were incubated without (control) or with 10 *μ*M CA. Hepatocytes were harvested at 36 h post transfection and processed for total RNA and protein extraction. To induce lipotoxicity and to establish the cell model, cells were incubated with 0.5 mM PA for 24 h.^47^

### Hepatic MDA content and serum levels of ALT, AST, TG, TC, LDL-C, HDL-C and SOD

Blood samples were obtained from the abdominal cavity and centrifuged at 3000 rpm for 15 min, and then serum was collected. The serum triglyceride (TG), total cholesterol (TC), alanine aminotransferase (ALT), aspartate aminotransferase (AST), low-density lipoprotein cholesterol (LDL-C) and high-density lipoprotein cholesterol (HDL-C) levels were measured according to the manufacturer's protocols (Nanjing Jiancheng Corp., Nanjing, China). MDA levels in the liver were quantified using a lipid peroxidation MDA assay kit (Beyotime Institute of Biotechnology, Jiangsu, China) according to the manufacturer's protocol. Hepatic SOD activity was determined using a SOD reagent kit (Nanjing Jiancheng Corp.). The final results were corrected for protein content.

### Liver histological examination

Paraffin-embedded liver tissue samples were cut into 5-*μ*m thick sections for hematoxylin and eosin staining, and the sections were then examined by light microscopy. Rat livers were fixed with 10% formalin, cut into 10-*μ*m sections with a cryostat for Sudan IV staining and then examined by light microscopy.

### TUNEL and caspase assays

Terminal deoxynucleotidyl transferase-mediated dUTP nick end labeling (TUNEL) staining was performed with an *In Situ* Cell Death Detection Kit (TMR Red; Roche, NJ, USA) according to the manufacturer's instructions. The nuclei were stained with DAPI. Caspase-3 and caspase-9 activity in the liver or cell lysates was measured using a caspase-3 or caspase-9 activity assay kit (Nanjing Jiancheng Corp.) according to the manufacturer's protocol.

### Quantitative RT-PCR

Total RNA was isolated with TRIZOL Reagent (TaKaRa, Dalian, China) according to the manufacturer's instructions. RNA samples typically had an A260/280 ratio between 1.9 and 2.1. The quantity and purity of the obtained total RNA samples were determined by UV spectroscopy (NanoDrop 2000 Spectrophotometer, Thermo Fisher Scientific, Waltham, MA, USA). Reverse transcription was performed with a TaqMan miRNA Reverse Transcription Kit, and mature miRNA was quantified by real-time PCR with a TaqMan miRNA assay Kit (GenePharma Corp.) using an Applied Biosystems 7300 System (Applied Biosystems, Foster City, CA, USA). miRNA expression was normalized to endogenous RNA U6 small nuclear 2 (RNU6B) expression.

### Western blotting analysis

Nuclear and cytosolic proteins were extracted from liver tissues with a protein extraction kit (KeyGen Biotech, Nanjing, China), and cells were lysed with RIPA buffer. Equal amounts of protein from each sample were separated by 10–15% SDS-PAGE (Bio-Rad, Hercules, USA). Blots were incubated overnight at 4 °C with the following primary antibodies: SIRT1 and p66shc (Abcam Ltd, Cambridge, UK); MnSOD, Bcl-xL and cleaved caspase-3 (Bioworld Technology, Inc., St Louis Park, MN, USA); and β-actin (Santa Cruz Biotechnology, Santa Cruz, CA, USA). The blots were then immunostained with secondary antibodies at 37 °C. The membranes were exposed to enhanced chemiluminescence-plus reagents (Beyotime Institute of Biotechnology). The images were captured using a BioSpectrum-410 multispectral imaging system and analyzed with Gel-Pro Analyzer (Version 5.0; Media Cybernetics, Rockville, MD, USA).

### Dual luciferase reporter assays

Plasmids containing the wild-type miR-34a-SIRT1 response element (wt-Luc-SIRT1) and the corresponding mutant (mut-Luc-SIRT1) were purchased from GenePharma Corp. (Shanghai, China). Plasmid DNA (wt-Luc-SIRT1, mut-Luc-SIRT1, or control vector) and ago-miR-34a or the ago-miR negative control were co-transfected into L02 cells. When appropriate, the cells were incubated with 10 μM CA or without CA (control) 24 h after transfection. Reporter assays were performed 36 h post transfection. Luciferase activity was measured with a Double-Luciferase Reporter Assay Kit (TransGen Biotec, Beijing, China) using the Dual-Light Chemiluminescent Reporter Gene Assay System (Berthold, Germany) and was normalized to Renilla luciferase activity.

### Statistical analyses

Statistical analyses were performed using GraphPad Prism (version 5.0; GraphPad Prism Software, La Jolla, CA, USA). The data were analyzed by a two-tailed unpaired Student's *t*-test or one-way analysis of variance to determine the statistical significance between the groups. Differences with *P*<0.05 were considered significant, and the results are presented as the mean±S.D.

## Abbreviations

NAFLD: nonalcoholic fatty liver disease
CA: carnosic acid
HFD: high-fat diet
ALT: alanine aminotransferase
AST: aspartate aminotransferase
TG: serum triglyceride
TC: total cholesterol
LDL-C: low-density lipoprotein cholesterol
HDL-C: high-density lipoprotein cholesterol
PA: palmitic acid
SOD: superoxide dismutase
MDA: malondialdehyde
MnSOD: manganese superoxide dismutase
Bcl-xL: B-cell lymphoma-extra large
